# Supplementary material for: Prevalence and Antimicrobial Resistance of Typhoid Fever in Ghana: A Systematic Review and Meta-Analysis
Source: Diseases. 2025 Apr 14;13(4):113. doi: 10.3390/diseases13040113 (PMC12025557; doi:10.3390/diseases13040113)
Supplement: Supplementary file 1 [file diseases-13-00113-s001.zip › diseases-3311065-supple/Supplemental_Table 1.docx]

**Table 1.** Quality assessment of studies based on the JBI checklist for prevalence studies.

| **Study ID** | **Was the sample frame appropriate to address the target population?** | **Were study participants sampled in an appropriate way** | **Was the sample size adequate** | **Were the study subjects and the setting described in detail** | **Was the data analysis conducted with sufficient coverage of the identified sample** | **Were valid methods used for the identification of the condition** | **Was the condition measured in a standard, reliable way for all participants** | **Was there appropriate statistical analysis** | **Was the response rate adequate, and if not, was the low response rate managed appropriately** | **Total** | **Grade** |
| --- | --- | --- | --- | --- | --- | --- | --- | --- | --- | --- | --- |
| Acquah et al., 2013 | 0 | 1 | 1 | 1 | 1 | 1 | 0 | 1 | 0 | **6** | Fair |
| Al-Emran et al., 2016 | 1 | 1 | 1 | 0 | 1 | 1 | 1 | 1 | 0 | **7** | Good |
| Anabire et al., 2018 | 1 | 1 | 1 | 1 | 1 | 1 | 0 | 1 | 0 | **7** | Good |
| Birkhold et al., 2023 | 1 | 1 | 0 | 0 | 0 | 1 | 1 | 0 | 0 | **4** | Fair |
| Domfeh et al., 2023 | 1 | 1 | 1 | 1 | 1 | 1 | 0 | 1 | 1 | **8** | Good |
| Eibach et al., 2016 | 0 | 1 | 1 | 1 | 1 | 1 | 1 | 0 | 0 | **6** | Fair |
| Eibach et al., 2016 | 1 | 1 | 1 | 1 | 0 | 1 | 1 | 0 | 1 | **7** | Good |
| Espinoza et al., 2016 | 0 | 1 | 1 | 1 | 1 | 1 | 1 | 1 | 1 | **8** | Good |
| Feglo et al., 2004 | 1 | 0 | 1 | 1 | 1 | 0 | 1 | 0 | 1 | **6** | Fair |
| Fusheini and Gyawu,2020 | 1 | 0 | 1 | 1 | 1 | 0 | 0 | 1 | 1 | **6** | Fair |
| Gross et al., 2011 | 0 | 1 | 1 | 0 | 0 | 1 | 1 | 0 | 1 | **5** | Fair |
| Koffuor et al., 2016 | 1 | 0 | 1 | 0 | 1 | 0 | 0 | 1 | 0 | **4** | Fair |
| Labi et al., 2014 | 1 | 0 | 1 | 1 | 1 | 1 | 1 | 1 | 1 | **8** | Fair |
| Marks et al., 2010 | 1 | 1 | 1 | 1 | 1 | 1 | 1 | 1 | 1 | **9** | Good |
| Marks et al., 2017 | 1 | 1 | 1 | 1 | 0 | 1 | 0 | 0 | 1 | **6** | Fair |
| Marks et al., 2024 | 1 | 1 | 1 | 1 | 1 | 1 | 1 | 1 | 1 | **9** | Good |
| Newman et al., 2011 | 1 | 1 | 1 | 0 | 1 | 1 | 1 | 1 | 1 | **8** | Good |
| Nielsen et al., 2012 | 1 | 1 | 1 | 1 | 1 | 1 | 1 | 1 | 1 | **9** | Good |
| Panzner et al., 2022 | 1 | 1 | 1 | 0 | 1 | 1 | 1 | 1 | 1 | **8** | Good |
| Rufai et al., 2023 | 1 | 1 | 1 | 1 | 1 | 1 | 1 | 1 | 1 | **9** | Good |
| Saana et al., 2014 | 1 | 1 | 1 | 0 | 1 | 1 | 1 | 1 | 1 | **8** | Good |
| Sothmann et al., 2015 | 1 | 1 | 1 | 1 | 1 | 1 | 1 | 1 | 1 | **9** | Good |

Yes- 1, No/ Unclear/ Not Applicable -0. Good - Low risk of bias, Fair, Moderate risk of bias, Poor – High risk of bias.

**Table 2.** Agreement of study selection across reviewers.

|  | **Reviewer 2** |  |  |
| --- | --- | --- | --- |
| **Reviewer 1** | **Included** | **Excluded** | **Sum** |
| Included | 17 | 4 | 21 |
| Excluded | 7 | 12 | 19 |
| Sum | 24 | 16 |  |

P_o_ = 29/40 = 0.725

P_e_ = 0.19 + 0.315 = 0.505.

Cohen’s kappa = (P_o_ - PE) / (1 - P_e_)

= (0.725- 0.505) / (1 – 0.505) = 0.44

Cohen’s kappa = 0.44.

P_o_ is the observed proportion of agreement.

P_e_ is the expected proportion of agreement by chance

**Table 3.** Interpretation of Cohen’s kappa.

| **Kappa** | **Level of Agreement** |
| --- | --- |
| > 0,8 | Almost perfect |
| > 0,6 | Substantial |
| > 0,4 | Moderate |
| > 0,2 | Fair |
| > 0 | Slight |
| < 0 | No agreement |
