# Supplementary figures and images for: Prevalence and Antimicrobial Resistance of Typhoid Fever in Ghana: A Systematic Review and Meta-Analysis
Source: Diseases. 2025 Apr 14;13(4):113. doi: 10.3390/diseases13040113 (PMC12025557; doi:10.3390/diseases13040113)

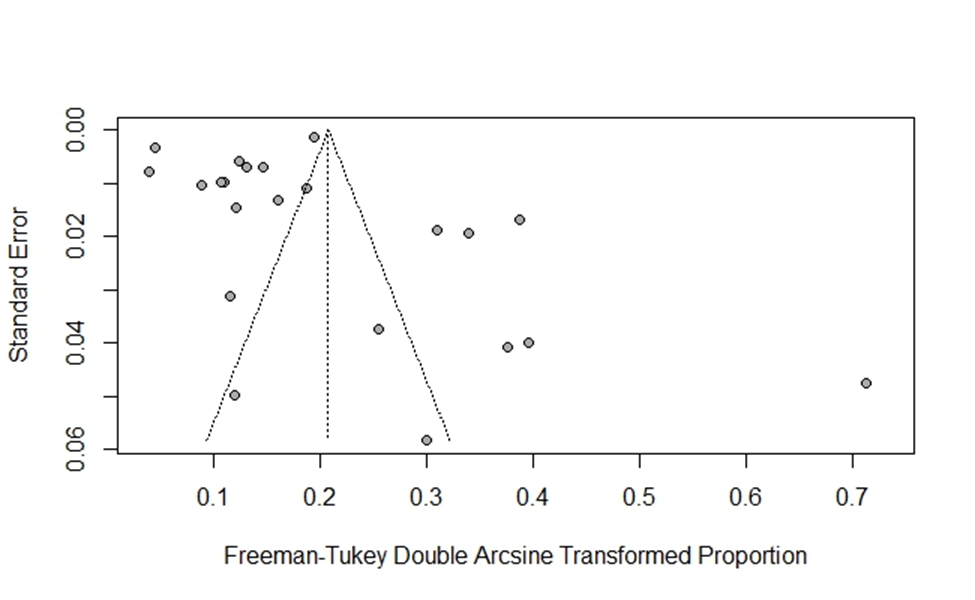


**Figure 1.** Funnel Plot distribution of included studies.

Supplement: Supplementary file 1 [file diseases-13-00113-s001.zip › diseases-3311065-supple/Supplemental_ Figure 1.docx]
